# A study on sensory properties of sodium reduction and replacement in Asian food using difference‐from – control test

**DOI:** 10.1002/fsn3.308

**Published:** 2015-11-01

**Authors:** Jasmine Leong, Chinatsu Kasamatsu, Evelyn Ong, Jia Tse Hoi, Mann Na Loong

**Affiliations:** ^1^ Singapore PolytechnicFood Innovation and Resource Centre500, Dover Road139651SingaporeSingapore; ^2^ Ajinomoto Co. Inc.TokyoJapan

**Keywords:** MSG, sensory analysis, sodium reduction, umami

## Abstract

This study examined the effects of sodium reduction and flavor enhancers on the sensory profile of two types of hawker foods commonly consumed in Singapore, namely chicken rice and *mee soto* broth. The ‘difference‐from‐control’ test was the method adopted in this study involving 24–29 trained panelists. Combinations included blind control, two levels of sodium reduction, and two levels of flavor enhancers in sodium‐reduced recipes. In the sodium‐reduced recipes, two levels of NaCl, 0.48% and 0.55%, for chicken rice, and 0.76% and 0.86% for *mee soto* (equivalent to 31% and 22% reduction in NaCl), were used. Monosodium glutamate (MSG) or *Ajiplus* ^®^ (a blend of MSG and nucleotides) at 0.20% and 0.40% were added to the recipes comprising a reduction of 40% in NaCl (equivalent to 31% and 22% reduction in sodium, respectively) compared with the control. It was found that the inclusion of MSG or *Ajiplus* ^®^ in 40% NaCl‐reduced recipe resulted in a significant increase in perception of umami taste (*P* < 0.05) when compared to the control. By adding flavor enhancers into the 40%‐reduced salt chicken rice recipes, the perception of saltiness was significantly increased when compared to 22% and 31% sodium reduced recipes. Similarly for *mee soto* broth, there was a significant increase in perception of chicken flavor, umami taste, mouthfeel sensation, and sweet taste (*P* < 0.05) with a decrease in the perception of sour and bitter taste when compared to control. By adding 0.40% MSG into the 40%‐reduced salt recipes, the perception of saltiness was maintained when compared with control.

## Introduction

High blood pressure or hypertension is a common condition that affects one in four adult Singaporeans (MOH, 2013). Risk factors that are known to contribute to high blood pressure (He and MacGregor 2010) include age, family history, stress, diet, activity level, and underlying diseases. It has been indicated that high salt intake is related to blood pressure increase which further increases the risk of heart disease and stroke. Thus, the reduction of dietary sodium intake is advisable to reduce risk in development of hypertension (MacGregor and de wardener 2002). According to the Health Promotion Board, current salt intake level in Singapore is 9 g per day, and this is significantly higher than the recommended 5 g per day or 2000 mg sodium per day (HPB, 2013a).

According to the National Nutrition Survey 2010 (MOH, 2011), 6 in 10 Singaporeans eat out at hawker centers at least once a day. Chicken rice, *mee soto*, fish ball noodles, fried carrot cake, mutton biriyani, and vegetarian fried beehoon are amongst the most heavily consumed hawker foods by Singaporeans and their respective sodium content per serving is high as surveyed by the Health Promotion Board (HPB, 2013b). Chicken rice is a meal composed of bite‐sized pieces of steamed chicken, fragrant rice, light, or dark soy sauce, a chilli, and ginger paste. The average sodium content of chicken rice is 1287 mg per serving, while the rice itself contains 820 mg. *Mee soto* is a spicy noodle soup dish served with shredded chicken. The average sodium content of *mee soto* is 2678 mg per serving, while the noodle itself contains 40 mg sodium. As the daily recommended intake of sodium is 2000 mg, one chicken rice meal would already meet 64.3% of the daily recommended intake; while a *mee soto* meal would exceed the daily recommended intake by about 33%.

Studies show that blood pressure can be lowered when intake of salt is reduced to less than 5 g per day (HPB, 2013a). This reduction of sodium intake from salt and other sources is able to lower blood pressure in both healthy individuals and individuals with high blood pressure. Therefore, the importance of sodium reduction is obvious. However, the main issue is that sodium‐reduced foods are often not palatable, thus general consumers tend to shy away from sodium‐reduced foods (Brady 2002). It is therefore pertinent to find ways of increasing the palatability for sodium‐reduced foods in order to increase the consumption rates of sodium reduced foods.

Food choices are made on the basis of taste, cost, and convenience, and, to a lesser extent, health, and variety (Glanz et al. 1998). Taste refers to the sensory appeal of foods, such as palatability, aroma, and texture. There are several reports outlining the use of glutamate salts such as monosodium glutamate (MSG) to enhance palatability of sodium‐reduced foods such as soup and broth (Chi and Chen 1992; Bellisle 1991; Carter et al. 2011). In particular, Ball et al. (2002), Roininen et al. (1996) and Yamaguchi (1987) had shown, that MSG could maintain food palatability with a lowered overall sodium level in a food when it was used to substitute some of the salt. According to Yamaguchi and Takashashi (1984), there is a compensative relationship between MSG and NaCl for maximum palatability. More MSG is needed when NaCl level is decreased and vice versa.

*AjiPlus* ^®^ (Ajinomoto Co., Inc., Tokyo, Japan) one of the umami seasoning was used in this study. It is made from the blending of MSG (glutamate) with nucleotides (sodium inosinate and guanylate). Glutamate is found naturally in foods such as meat, seafood, vegetables, cheese, and milk. It is not a salt taste replacer but imparts a savory taste called “umami” which enhances the palatability of foods (Yamaguchi 1987; Yamaguchi and Ninomiya 2000; Halpern 2002; Jinap and Hajeb 2010). Inosinate is found in fish, beef, and shrimp. Guanylate is found in particularly high quantities in shiitake mushroom which accounts for its strong umami taste (Reed and Nagodawithana 1991).

The aim of this study was to apply the sensory test of difference‐from‐control to determine the effect of salt reduction and the use of MSG and nucleotides on the sensory profile of chicken rice and *mee soto* broth.

## Materials and Methods

### Sample preparation

#### Chicken rice

Four batches of the recipe were made into paste that would later be used to cook the chicken rice (see Table 1). Each batch yielded 4.95 kg paste from 5.50 kg of starting ingredient due to water loss which resulted in concentration of the product. All four batches of paste, weighing 19.80 kg in total, were mixed together before separating into seven portions. Six portions of paste were subsequently mixed with three levels of sodium chloride (0.42%, 0.48%, and 0.55% which were equivalent to 40%, 31%, and 22% reduction in sodium chloride, respectively), MSG or *AjiPlus* ^®^ (0.20% and 0.40%). One portion was added with 0.70% salt without flavor enhancers as the control. See Table 2 on experimental design for this study.

**Table 1:** Composition of ingredients used in the base recipes of chicken rice paste and *mee soto* broth

IngredientsChicken rice paste	Quantity (%)	Ingredients*Mee Soto* broth	Quantity (%)
Vegetable oil	29.35	Vegetable oil	8.38
Young ginger	18.00	Shallot	33.50
Shallot	17.00	Garlic	5.56
Garlic	17.00	Red onion	19.54
Sugar	5.00	Candlenuts	3.35
Chicken flavor	0.25	Galangal	6.98
Lemongrass flavor	0.10	Lemongrass	8.38
Pandan flavor	0.20	Sugar	3.23
Water	12.90	Ground cumin	0.56
Xanthan Gum	0.20	Ground coriander	1.12
		Fennel seeds	0.56
		Ground turmeric	0.11
		White pepper	0.98
		Chicken flavor	1.28
		Water	6.47
Total	100.00	Total	100.00

**Table 2:** Experimental design for the study of reduced salt chicken rice and *mee soto* broth

Control^1^
22% NaCl reduction
31% NaCl reduction
40% NaCl reduction + 0.20% *Ajiplus* ^®^
40% NaCl reduction + 0.20% MSG
40% NaCl reduction + 0.40% *Ajiplus* ^®^
40% NaCl reduction + 0.40% MSG

^1^Control = no sodium reduction (NaCl_chicken rice_ = 0.70% and NaCl_mee soto broth_ = 1.10%).

After addition to the flavor enhancers at dosages of 0.20% and 0.40% of either MSG or *AjiPlus* ^®^, the content of each portion was thoroughly mixed to ensure a homogenous distribution of the added ingredients. The targeted reduction in sodium content ranged from 22% to 31% with reference to control formulation (Table 1). The samples were then packed into labeled nylon bags in 80–82 g portions and stored at −20°C.

The day before sensory evaluation, the batches of paste were removed from freezer and left at 4°C to thaw overnight. The paste, weighing about 75 g was cooked together with 300 g of rice and 570 g of water in a Philips rice cooker (model no. HD 4755/00, Philips Singapore, Singapore), using the setting for regular plain rice. Once the chicken rice was cooked, about 50 g of rice was weighed and portioned into a serving cup and left on the plastic perforated steam tray (21 cm in diameter) in the rice cooker until they were ready to serve at 70°C. This was to ensure that all the samples were served at the same temperature.

#### *Mee soto* broth

Seven batches of the recipe were cooked into a paste that would later be used to cook the *mee soto* broth. Each batch yielded 5.65 kg paste from 6.70 kg of starting ingredients due to water loss which resulted in concentration of the product. All seven batches of paste, weighing 39.55 kg in total, were mixed together before separating into seven portions. Six portions of paste were subsequently mixed with different amounts of sodium chloride (0.66%, 0.76%, and 0.86% which were equivalent to 40%, 31%, and 22% reduction in sodium chloride, respectively), MSG or *Ajiplus* ^®^ (0.20% and 0.40%). One portion was added with 1.10% salt without flavor enhancers as the control. See Table 2 on experimental design for this study. After addition of flavor enhancers, the content of each portion was thoroughly mixed to ensure a homogenous distribution of the added ingredients. The targeted reduction in the sodium content ranged from 25% to 35% with reference to control formulation. The samples were then packed into labeled nylon bags in 222 g portions and stored at −20°C.

The day before sensory evaluation, a number of control and samples were removed from the freezer and left at 4°C to thaw overnight. The pastes were cooked together with water in a Philips rice cooker (HD 4755/00), using the setting for soup. After 10 minutes of cooking, the soup was strained and the rice cooker was set to keep warm mode (74.5 ± 0.4°C). The broth was portioned into 35 g serving cups and left on the plastic perforated steam tray in the rice cooker until they were ready to serve at 70°C. This was to ensure that all the samples were served at the same temperature.

### Sensory evaluation

#### Training of panels

A total of 44 staff members of Singapore Polytechnic (SP), aged between 21 years and 60 years, participated in the sensory screening tests. All of the panelists were untrained when recruited. Prior to participation, the panelists were screened on their ability to identify the five basic tastes (sweet, salty, sour, bitter, and umami). Panellists were given six samples namely, water and solutions of sugar (16 g/L), sodium chloride (5 g/L), caffeine (0.5 g/L), citric acid (1 g/L), and monosodium glutamate (1.9 g/L). In addition, the panelists were presented with a series of coded salt solutions with concentrations of 0.40%, 0.60%, 0.80%, 1.00%, and 1.50% (w/v) and asked to rank the solutions in order from saltiest to least.

A total of 29 panelists were selected after the screening process. Under the direction of the panel leader, the panelists were then asked to generate descriptors that well defined the two products. Through consensus, panelists generated a set of agreed terms that describe differences amongst the products. Descriptors were referenced and all panelists were trained on those descriptors (Table 3).

**Table 3:** Definitions of the sensory attributes of chicken rice and *mee soto* broth developed by the trained panelists during training

Sensory attribute	Interpretation
Chicken rice	
Overall flavor	Overall flavor associated with cooked chicken rice^1^
Chicken flavor	Sensations associated with cooked chicken^1^
Herbs/spices flavor	Sensations associated with garlic, shallot, ginger and pandan leaf^1^
Salty	Taste on the tongue associated with sodium chloride^2^
Umami	Taste on the tongue associated with monosodium glutamate^2^
Mouthfeel	Full flavor sensation in the mouth^1^
Mee soto broth	
Overall flavor	Overall flavor associated with cooked mee soto broth^1^
Chicken flavor	Sensations associated with cooked chicken^1^
Herbs/spices flavor	Sensations associated with garlic, shallot, ginger and pandan leaf^1^
Salty	Taste on the tongue associated with sodium chloride^2^
Umami	Taste on the tongue associated with monosodium glutamate^2^
Mouthfeel	Full flavor sensation in the mouth^1^
Sweet	Taste on the tongue associated with sucrose^2^
Sour	Taste on the tongue associated with citric acid^2^
Bitter	Taste on the tongue associated with caffeine^2^

^1^Definitions as developed by the panelists.

^2^Definitions of Meilgarrd et al. (2007).

#### Tasting procedure

The trained panel testing was conducted in the sensory laboratory at Food Innovation & Resource Centre (FIRC) at the Singapore Polytechnic. The requirements of ISO8589 (2010) in the aspects of individual booths, standard lightings, and temperature were fulfilled. Directional difference from control test (DFC) was conducted to statistically determine if a perceptual sensory difference exists between the control and one or more test samples and to estimate the degree of the difference, if one exists.

A total of 29 panelists were selected for the sensory evaluation of the chicken rice. As for *mee soto* broth, there were 24 panelists participated in the study. Both sets of panelists participated in six training sessions, followed by six taste‐testing sessions. Each training sessions took place for 1.5 h. During training, panelists were given references that were associated with the sensory attributes of the chicken rice and *mee soto*. They became more confident with the scoring of the sensory attributes by having the samples presented at least two times per session to allow refamiliarization with the typical flavor/taste associated with each attribute.

For each taste‐testing session of chicken rice, each panelist was presented with three sets of DFC samples of 50 g each. As for *mee soto* broth, each sample was about 35 g. One of these sets was a comparison between a control and a blind control while the other two sets were a comparison between a control and one of the six reduced sodium samples. Each low‐sodium sample and the three blind controls were presented to panelists, using a three‐digit coded sample while the control sample was presented as “Control” in three separate sessions with randomised order. All the tests were conducted in duplicate. The three blind controls were used to check the homogeneity of the sample preparation and to monitor the performance of the panel. The panelists were requested to assess each coded sample, comparing it to the control (C), and to assess the degree of difference using a 11‐point scale where 1 = much less intense than control, 6 = no difference from control, 11 = extremely intense than control. Ethics approval from Singapore Polytechnic Ethics Review Committee and written informed consent from all subjects were obtained before the commencement of the experiment.

### Sodium analysis of samples

All samples were analyzed for sodium, using the acid digestion/ICP‐OES method (AOAC 984.27).

### Data analysis

Sensory analysis was repeated twice in completely randomized blocks. Significance of the results was tested using analysis of variance (ANOVA). The Dunnett's Test was applied to compare each treatment mean to control treatment mean using the SPSS software ver. 18 (SPSS, Inc., Chicago, IL)

In order to find out if there is a significant difference between two treatments, the Thurstonian model was used where the Thurstonian *δ* was obtained (Bi 2008). Sensory difference in terms of *δ* can be estimated from the observed proportion of correct responses in different discrimination methods. The estimate of *δ* is denoted by *d′*. The precision of the estimate *d′* can be expressed by its variance, *V*(*d′*). In this study, difference from control was used where each treatment was compared with the control. If the panelists denoted a ‘6’ on the scale for comparison between different treatments and control, the score will be ‘0’ which indicated as not different from control; ‘−1’ as weaker than control and ‘1’ as stronger than control. The number of correct responses was summed up and the *d′* and *B* values obtained from the Table of *d′* and *B* values for 2‐AFC method. The variance, *V*(*d′*) which is the quotient between *B* and *N* (sample size) was then obtained.

The test statistic *Z* was used for the comparison of *d'*s:

$$Z=\frac{d_{1}^{\prime }-d_{2}^{\prime }}{\sqrt{V\left(d_{1}^{\prime }\right)+V\left(d_{2}^{\prime }\right)}}$$

If |*Z*| > *z*_1 − *α*/2,_ the conclusion is that the two *d'*s are significantly different at an *α* significance level, where *α* = 5%.

## Results

### Sensory evaluation

A Dunnett's test for multiple comparisons with a control was applied to the means of the reduced sodium samples. To determine if there was a significant difference between two treatments, the Thurstonian model was used where the Thurstonian *δ* was obtained.

There was no significant difference among the three blind controls, which indicated good homogeneity in the sample preparation for both chicken rice and *mee soto* broth.

#### Chicken rice

It was revealed in Table 4 that the sample with 40% NaCl reduction plus 0.40% MSG or *Ajiplus* ^®^ was significantly more intense in overall flavor intensity compared to the control. In addition, samples with 0.40% of flavor enhancers were more intense in chicken flavor compared to those with 0.20% or without flavor enhancers when compared to control. The 22% and 31% NaCl reduction sample was significantly lower in chicken flavor compared to control. As shown in Table 5, there was a no significant difference in overall flavor and chicken flavor between 40% reduced salt recipes with flavor enhancers and those with 22% and 31% reduced salt recipes.

**Table 4:** Mean values degree of difference from control for attributes in chicken rice with different levels of sodium and flavor enhancers by trained panelists^2^ using Dunnertt's test

Sample	Attributes^3^,^4^					
	Overall flavor	Chicken flavor	Herb/spice flavor	Saltiness	Umami	Mouthfeel
Blind control^1^	5.57	5.98	5.89	6.06	5.98	5.93
22% NaCl reduction	5.17	5.14*	5.10*	4.31*	5.05*	4.91*
31% NaCl reduction	5.81	4.53*	5.18*	3.95*	4.51*	4.67*
40% NaCl reduction + 0.20% *Ajiplus* ^®^	6.16	6.17	6.05	4.91*	7.19*	6.50
40% NaCl reduction + 0.20% MSG	5.69	6.03	6.00	4.86*	6.79*	6.02
40% NaCl reduction + 0.40% *Ajiplus* ^®^	6.17*	6.71*	6.28	5.57	7.98*	7.28*
40% NaCl reduction + 0.40% MSG	6.12*	6.62*	6.24	5.45	7.95*	7.21*
*P* value	<0.05	<0.001	<0.001	<0.001	<0.001	<0.001

^1^Blind control = no sodium reduction (NaCl = 0.70%); no significant difference among the blind controls.

^2^Mean obtained through 2 repetitions by 29 panelists each.

^3^Mean on the vertical marked with * differed significantly from control (Dunnett's Test, *P* = 0.05%).

^4^Score ranges from 1 = much less intense than control, 6 = no difference from control, 11 = extremely intense than control.

**Table 5:** *Z*‐values for the comparison of Thurstonian d' values between chicken rice samples with 22% and 31% NaCl reduction with flavor enhancer (MSG or *Ajiplus* ^®^) vs. 40% NaCl reduction with two levels of MSG and *Ajiplus* ^®^ (0.20% and 0.40%)

Attribute	122% reduction NaCl vs. 40% reduction NaCl +		231% reduction NaCl vs. 40% reduction NaCl +	
	0.40% MSG	0.40% *Ajiplus* ^®^	0.20% MSG	0.20% *Ajiplus* ^®^
Flavor intensity	n.a.	n.a.	n.a.	n.a.
Chicken flavor	n.a.	n.a.	n.a.	n.a.
Herb/Spice flavor	n.a.	n.a.	n.a.	n.a.
Salty	2.442*	3.152*	2.656*	2.656*
Umami	−2.845*	−2.622*	0.781	−0.617
Mouth feel	n.a.	n.a.	n.a.	n.a.

^1^Average sodium concentration of these groups of recipes = 213 ppm.

^2^Average sodium concentration of these groups of recipes = 193 ppm.

*Denotes significant difference at *α* = 5% where |*Z*| > 1.96 between samples of 21% and 31% NaCl reduction without flavor enhancer (MSG or *Ajiplus* ^®^) vs. 40% NaCl reduction with two levels of MSG or *Ajiplus* ^®^ (0.20% and 0.40%).

n.a. denotes a *z* value that cannot be calculated as the observed proportion of correct responses falls below 50.

Samples without flavor enhancers were significantly less intense (*P* < 0.05) in herb/spice flavor compared to control (Table 4). The rest of the recipes with flavor enhancers were higher in herb/spice flavor but not significant when compared to control. All samples were significantly lower in saltiness than control except for the reduced salt recipes with 0.40% flavor enhancers that were slightly lower than control. Recipes with 40% reduced salt plus 0.20% flavor enhancers and those with 31% and 22% reduced salt were perceived less salty when compared to those with 40% reduced salt plus 0.40% flavor enhancers (Table 4). There was a significant difference in saltiness between 40% reduced salt recipes with flavor enhancers (*z* > 1.96) and those with 22% and 31% reduced salt recipes (Table 5).

The addition of flavor enhancers to reduced salt recipes led to a greater intensity in umami taste and mouthfeel compared with control (Table 4). All samples with 0.40% flavor enhancers were significantly higher in umami and mouthfeel intensity when compared with control (*P* < 0.05). Recipes with 31% and 22% reduced salt were perceived having less umami when compared to those with 40% reduced salt plus 0.20% and 0.40% flavor enhancers (Table 4). In addition, there was a significant difference in umami between 40% reduced salt recipes with 0.40% flavor enhancers [0.40% MSG (*z* = −2.845) and 0.40% *Ajiplus* (*z* = −2.622)] and those with 22% reduced salt recipes (Table 5) but this difference was not observed between 40% reduced salt recipes with 0.20% flavor enhancers and those with 31% reduced salt recipes.

No significant difference was observed in all attributes between 22% reduced salt recipes and 31% reduced salt recipes (not shown in table). Similarly, there was no significant difference in saltiness, umami and mouthfeel between recipes with 0.40% and 0.20% MSG and those with 0.40% and 0.20% *Ajiplus*, respectively (Table 6).

**Table 6:** *Z*‐values for the comparison of Thurstonian d' values between chicken rice samples with 40% NaCl reduction with 0.20% and 0.40% MSG vs. those with 40% NaCl reduction with 0.20% and 0.40% *Ajiplus* ^®^

Attribute	40% reduction NaCl+	
	10.40% MSG vs. 0.40% *Ajiplus* ^®^	20.20% MSG vs. 0.20% *Ajiplus* ^®^
Flavor intensity	n.a.	n.a.
Chicken flavor	n.a.	n.a.
Herb/Spice flavor	n.a.	n.a.
Salty	0.7499	0.0000
Umami	0.2411	−1.3937
Mouth feel	−0.3956	n.a.

^1^Average sodium concentration of these groups of recipes = 217 ppm.

^2^Average sodium concentration of these groups of recipes = 194 ppm.

*Denotes significant difference at *α* = 5% where |*Z*| > 1.96 between samples with 40% NaCl reduction with 0.20% and 0.40% MSG vs. those with 40% NaCl reduction with 0.20% and 0.40% *Ajiplus* ^®.^

n.a. denotes a *z* value that cannot be calculated as the observed proportion of correct responses falls below 50.

#### *Mee Soto* broth

From Table 7, the overall flavor intensity in *mee soto* broth rose significantly with the inclusion of 0.40% *Ajiplus* ^®^ in the reduced salt sample. The addition of flavor enhancers generally increased the flavor intensity of *mee soto* although only the difference in 0.40% *Ajiplus* ^®^ recipe was significant. Furthermore, the addition of MSG and *Ajiplus* ^®^ in 40% NaCl reduction samples led to higher chicken flavor intensity significantly as compared with control.

**Table 7:** Mean values degree of difference from control for attributes in *mee soto* broth with different levels of sodium and flavor enhancers by trained panelists^2^ using Dunnertt's test

Sample	Attributes^3^								
	Overall flavor	Chicken flavor	Herb/spice flavor	Saltiness	Umami	Sweet	Sour	Bitter	Mouthfeel
Blind control^1^	5.87	6.03	6.25	6.16	6.04	5.97	6.10	5.94	6.05
22% NaCl reduction	6.13	6.13	6.35	5.08*	6.38	6.29	5.44*	5.60	6.00
31% NaCl reduction	6.10	6.17	6.38	4.50*	6.19	6.34	5.21*	5.63	5.94
40% NaCl reduction + 0.20% *Ajiplus* ^®^	6.25	6.70*	6.32	4.87*	7.39*	6.89*	4.73*	4.85*	7.13*
40% NaCl reduction + 0.20% MSG	6.08	6.99*	6.29	4.89*	7.25*	7.18*	4.67*	4.50*	7.25*
40% NaCl reduction +0.40% *Ajiplus* ^®^	6.63*	7.77*	6.06	4.98*	7.85*	7.79*	4.54*	4.50*	7.65*
40% NaCl reduction + 0.40% MSG	6.31	7.85*	6.52	5.47	7.79*	7.77*	4.57*	4.33*	7.81*
*P* value	<0.05	<0.001	0.853	<0.001	<0.001	<0.001	<0.001	<0.001	<0.001

^1^Blind control = no sodium reduction (NaCl = 1.10%); no significant difference among the blind controls.

^2^Mean obtained through 2 repetitions by 24 panelists each.

^3^Mean on the vertical marked with * differed significantly from control (Dunnett's Test, *P* = 0.05%).

Score ranges from 1 = much less intense than control, 6 = no difference from control, 11 = extremely intense than control.

There was no significant difference between control and all samples with respect to herb/spice flavor (Table 7). Saltiness of all samples was significantly lower compared to the control, except reduced salt recipe with 0.40% MSG which was slightly lower than control. There was no significant difference in saltiness between 40% reduced salt recipes with flavor enhancers and those with 22% and 31% reduced salt recipes (Table 8).

**Table 8:** *Z*‐values for the comparison of Thurstonian d' values between *mee soto* broth samples with 22% and 31% NaCl reduction with flavor enhancer (MSG or *Ajiplus* ^®^) vs. 40% NaCl reduction with two levels of MSG and *Ajiplus* ^®^ (0.20% and 0.40%)

Attribute	122% reduction NaCl vs. 40% reduction NaCl +		231% reduction NaCl vs. 40% reduction NaCl +	
	0.40% MSG	0.40% *Ajiplus* ^®^	0.20% MSG	0.20% *Ajiplus* ^®^
Flavor intensity	n.a.	n.a.	n.a.	n.a.
Chicken flavor	n.a.	n.a.	n.a.	n.a.
Herb/Spice flavor	n.a.	n.a.	n.a.	n.a.
Salty	0.506	0.080	1.587	1.162
Umami	n.a.	n.a.	n.a.	n.a.
Sweet	n.a.	n.a.	−4.096*	−2.669*
Sour	n.a.	n.a.	−2.105*	−0.617
Bitter	n.a.	n.a.	n.a.	n.a.
Mouth feel	n.a.	n.a.	n.a.	n.a.

^1^Average sodium concentration of these groups of recipes = 357 ppm.

^2^Average sodium concentration of these groups of recipes = 311 ppm.

*Denotes significant difference at *α* = 5% where |*Z*| > 1.96 between samples of 21% and 31% NaCl reduction without flavor enhancers (MSG or *Ajiplus* ^®^) vs. 40% NaCl reduction with two levels of MSG or *Ajiplus* ^®^ (0.20% and 0.40%).

n.a. denotes a *z* value that cannot be calculated as the observed proportion of correct responses falls below 50.

Similarly, the inclusion of 0.40% flavor enhancers to reduced salt recipes significantly increased the intensity in umami, sweetness and mouthfeel compared to control (Table 7). The umami, sweet, and mouthfeel intensities were higher in recipes with 0.40% flavor enhancers than those with 0.20% when compared with control. In contrast, the samples containing 0.20% and 0.40% flavor enhancers significantly reduced in sourness and bitterness compared to control (Table 7). There was a significant difference in sweetness between 40% reduced salt recipes with 0.20% flavor enhancers [0.20% MSG (*z* = −4.096) and 0.20% *Ajiplus* (*z* = −2.669)] and those with 31% reduced salt recipes (Table 8). The difference in sourness was only significant between 40% reduced salt recipes with 0.20% MSG and those with 31% reduced salt recipes (*z* = −2.105).

No significant difference was observed in all attributes between 22% reduced salt recipes and 31% reduced salt recipes (not shown in table). Similarly, there was no significant difference in almost all the attributes between recipes with 0.40% and 0.20% MSG and those with 0.40% and 0.20% *Ajiplus* ^®^, respectively (Table 9).

**Table 9:** *Z*‐values for the comparison of Thurstonian d' values between *mee soto* broth samples with 40% NaCl reduction with 0.20% and 0.40% MSG vs. those with 40% NaCl reduction with 0.20% and 0.40% *Ajiplus* ^®^

Attribute	40% reduction NaCl +	
	10.40% MSG vs. 0.40% *Ajiplus* ^®^	20.20% MSG vs. 0.20% *Ajiplus* ^®^
Flavor intensity	n.a.	n.a.
Chicken flavor	0.5362	1.0449
Herb/Spice flavor	n.a.	0.8347
Salty	0.0801	−0.4330
Umami	0.0000	−0.2673
Sweet	0.5781	1.7252
Sour	−0.3019	0.2270
Bitter	0.2391	0.8805
Mouth feel	0.0000	1.5050

^1^Average sodium concentration of these groups of recipes = 354 ppm.

^2^Average sodium concentration of these groups of recipes = 308 ppm.

*Denotes significant difference at *α* = 5% where |*Z*| > 1.96 between samples with 40% NaCl reduction with 0.20% and 0.40% MSG vs. those with 40% NaCl reduction with 0.20% and 0.40% *Ajiplus* ^®.^

n.a. denotes a *z* value that cannot be calculated as the observed proportion of correct responses falls below 50.

### Sodium analysis

The results in Table 10 indicated that there was a reduction of sodium ranging from 27% to 36% and 24% to 36% for chicken rice and *mee soto* broth, respectively. Reduction of NaCl to 40% with the addition of 0.20% flavor enhancers seemed to show similar level in sodium reduction (approximately 35% reduction in sodium) as compared with 31% NaCl reduction recipe. Similar trend in sodium reduction (approximately 29%) was observed between the recipe of 40% NaCl reduction plus 0.40% flavor enhancers and 22% NaCl reduction recipe.

**Table 10:** Sodium content of chicken rice and *Mee soto* broth

	Chicken rice		*Mee soto* broth	
	Sodium content (ppm)	Sodium reduction (%)	Sodium content (ppm)	Sodium reduction (%)
Blind Control^1^	299	–	475	–
22% NaCl reduction	206	31	363	24
31% NaCl reduction	192	36	319	33
40% NaCl reduction + 0.20% *Ajiplus* ^®^	197	34	306	36
40% NaCl reduction + 0.20% MSG	191	36	309	35
40% NaCl reduction + 0.40% *Ajiplus* ^®^	218	27	358	25
40% NaCl reduction + 0.40% MSG	215	28	349	27

^1^Blind control = no sodium reduction (NaCl = 0.70% and 1.10% for chicken rice and *Mee soto*, respectively); no significant difference among the blind control.

## Discussions

Taste is one of the most important factors that influence the buying decision of consumers (Drewnowski & Darmon, 2005; Glanz et al. 1998). Sodium improves the taste and other sensory attributes of food products. In Singapore, eight in ten Singapore residents exceeded the daily salt intake recommendation of less than 5 g/day. In the National Health Survey in 2010 (MOH, 2011), it was found that the individual's daily salt intake was 8.3 g which is more than 60% above the recommended level. Excessive intake of salt is a main risk factor for high blood pressure. Scientific studies have provided strong evidence that lowering sodium intake is beneficial in reducing blood pressure. HPB has been working with food industry partners to develop a ‘healthier salt’ and other foods with lower sodium content. The Healthier Choice Symbol (HCS) by HPB has 25% less sodium compared to regular salt content in the food. However, the goal to reduce salt while maintaining the palatability of the food could be quite challenging and hence, the possible use of flavor enhancers to address this issue was studied. In this present study of two savory meals, the amount of sodium reduction was more than 25%. It was found that in chicken rice attributes such as overall flavor, chicken flavor and umami were more intense except for saltiness when compared with control. In terms of overall flavor, chicken flavor and umami, the same finding applied to *mee soto* broth. These attributes were more intense except for saltiness, sour, and bitter taste. This is in agreement with a study by Yamaguchi (1998) and Yamaguchi and Takashashi (1984) who stated that umami substances are effective flavor enhancers in savory foods, such as meat, fish, seafood, vegetable foods and mixed products.

Soups like *mee soto* belong to those foods in which added umami substances resulted in positive palatability changes (Baryłko‐Pikielna and Kostyra 2007). Thus, they are quite commonly used as test material in the experimental studies on the effect of MSG or MSG and nucleotides on palatability test. Roininen et al. (1996) studied the effect of umami substances, MSG (0.20%), and IMP and GMP (0.05%), on the acceptance of three low‐salt soups (lentil and mushroom, leek‐potato and minestrone) in two groups of subjects with low‐salt and high‐salt preference. Baryłko‐Pikielna and Kostyra (2007) studied the palatability changes in six types of soup evoked by various combinations of MSG (0%, 0.10%, 0.30%, 0.50%) and nucleotides (0%, 0.005%, 0.01%, 0.015%).

In the present study, the levels of flavor enhancers used were 0.20% and 0.40% and the level of salt ranged from 0.42% to 0.55% for chicken rice; and 0.66% to 0.86% for mee soto broth. These corresponded to 22% to 40% reduction in NaCl. Yamaguchi and Takashashi (1984) used an optimal level of 0.38% and 0.81% in MSG and NaCl in soup, respectively. According to the authors there was a compensative relationship between the MSG and NaCl for maximum palatability; more MSG was needed when the NaCl level decreased and vice versa. By adding MSG appropriately, the sodium chloride addition could be reduced by 30–40% while maintaining the same palatability, although perception of saltiness was reduced (Yamaguchi and Takashashi 1984). In addition, Ball et al. (2002) showed that addition of MSG or calcium diglutamate (CDG) in sodium reduced recipe of pumpkin soup increased the liking rating while the saltiness rating decreased compared to the reference soup. Carter et al. (2011) showed that addition of CDG does not affect salty rating of chicken broth.

The current study has shown that by adding 0.40% flavor enhancers into the 40% reduced salt recipes of chicken rice and 0.40% MSG into the 40% reduced salt recipe of *mee soto* broth, the perception of saltiness was maintained when compared with those without flavor enhancers (control). Furthermore, saltiness in 40% reduced salt recipes of chicken rice with 0.40% flavor enhancers is significantly higher when compared with 22% reduced salt recipe although their sodium level is the same. However, this saltiness enhancement was not observed in *mee soto* broth. According to the report by Carter et al.(2011 *)*, there was a tendency that the perception of saltiness increased when NaCl concentration is low (0.16% and 0.53% w/w NaCl) but not when NaCl concentration is high (0.85% and 1.70% w/w NaCl). Therefore, it can be assumed that saltiness enhancement was not observed in *mee soto* broth because NaCl concentration is higher (0.86%) than that of chicken rice (0.55%).

Several studies have been established that an addition of MSG to soups and other foods could result in the use of lesser amount of added NaCl (Yamaguchi and Takashashi 1984; Chi and Chen 1992 and Altug and Demirag 1993). The potential reduction of salt in soups was shown to be up to approximately 40% that corresponds to 12% sodium reduction (Altug and Demirag 1993). From the results in sodium content of chicken rice and *mee soto* broth, partial replacement of table salt by MSG allowed substantial reduction of Na intake between 25% and 36%, which met the Healthier Choice Symbol requirement in Singapore.

In the present study of chicken rice, addition of 0.40% flavor enhancers to reduced salt recipe resulted in a significant increase in umami and mouthfeel intensity as compared to recipes with reduced salt only. As for the *mee soto* broth, the chicken flavor, umami, sweetness, and mouthfeel intensity increased significantly when flavor enhancers were used in the reduced salt recipes. MSG does not necessarily have positive effects on all types of foods but a combination of NaCl and MSG at the adequate concentrations and ratio to each other with nucleotides and amino acids may result in enhanced palatability (Yamaguchi 1998).

It is also shown in the present study that recipes with higher salt content exhibited increased perception of umami among the panelists. This can be supported by Carter et al. (2011) who showed the effect of NaCl concentration from 0.16% to 1.70% in chicken broth on the savory intensity score (also known as umami) which was increased almost by three fold; while the hedonic score increased close to two fold. Kim and Kim (2014) demonstrated using a taste sensing system that umami increased with a saltiness score (*r* = 0.624) in a Korean Makgeolli, which is an alcoholic rice beverage. A positive relationship between salt concentration and umami was also demonstrated in a study of salt replacement in Bouillon cubes by Batenburg et al. (2010). In a similar way, Møller et al. (2013) studied the effects of salt on sensory characteristics of Cheddar cheese and concluded that umami taste increased with the increasing concentration of NaCl (from 0.9% to 2.3% wt/wt) in the cheese.

There was a tendency to increase the overall flavor, chicken flavor and umami in the reduced salt chicken rice recipes with *Ajiplus* ^®^ compared to those with MSG as shown in Table 4. This was also observed in the *mee soto* broth recipe in which the overall flavor and umami taste were tend to be higher in intensity compared to those with MSG. In addition, the overall flavor in recipe with 0.40% *Ajiplus* ^®^ was significantly higher than that in recipe with 0.40% MSG. And saltiness in recipe with 0.40% MSG was significantly higher than that in recipe with 0.40% *Ajiplus* ^®^. However, there was no significant difference in all attribute in chicken rice and *mee soto* broth between recipes with *Ajiplus* ^®^ and those with MSG (Table 6). It shows that the combination of nucleotides with MSG has synergistic effect and this is in agreement with the studies conducted by Rifkin and Bartoshuk (1980) and Yamaguchi (1967). The phenomenon of synergism is of great importance as this unique function of nucleotide as flavor enhancers provides an opportunity for the food industry to use lesser MSG in the formulation without affecting flavor quality. However, the levels of nucleotides used are likely to change, depending on the type of food application and the specific flavor enhancer selected.

While MSG and *Ajiplus* ^®^ helped to enhance the intensity of herb/spice flavor in chicken rice, this was not evident in *mee soto* broth. There were more herbs and spices used in *mee soto* broth and thus the subtle differences may not be detected by the panelists as the overall flavor may be overpowering.

In *mee soto* broth, sour and bitter tastes were significantly lower in those recipes with flavor enhancers compared to control. This indicated that flavor enhancers could have reduced the bitter and sour taste. It has been known that MSG can supress sour and bitter taste (Yamaguchi 1998). Woskow (1969) has shown in a study that flavor enhancing nucleotides like IMP and GMP can suppress some bitter and sour notes, but enhance sweet and salt perceptions. The concept of suppression of undesirable flavor using different flavor enhancers of different concentrations could be further investigated with other interesting food matrix.

## Conclusions

It is feasible to add some MSG and nucleotides to foods to allow reduction in the amount of added NaCl. In this study, partial replacement of table salt by MSG or *Ajiplus* ^®^ (mixture of MSG and nucleotides) (0.20% and 0.40%) allowed substantial reduction of sodium intake, without reducing flavor and taste. The reduction of sodium content resulted from lowering of NaCl level (22%, 31% and 40%), despite the addition of some sodium in MSG. The potential reduction of sodium in two popular dishes in Singapore – chicken rice and *mee soto* broth ranged from 24% to 36% (based on analysis). The reduced salt samples without any addition of MSG or *AjiPlus* ^®^ produced consistently lower values for chicken flavor, herbs/spices flavor, salty taste, umami taste, and mouthfeel sensation in chicken rice. Similarly, in *mee soto* broth, the intensity of chicken flavor, umami taste, sweet taste, and mouthfeel were decreased in the reduced salt samples without any addition of MSG or *AjiPlus* ^®^ were observed.

By adding flavor enhancers into the 40% reduced salt recipes, the perception of saltiness was enhanced when compared with those without flavor enhancers in chicken rice. It has also been shown that MSG has a positive effect on the flavor intensity, umami, and mouthfeel of these foods. The palatability test can be conducted, using the consumer panel to evaluate the degree of liking for these reduced salt recipes.

## Conflict of Interest

None declared.
